# Closed-loop bioelectronic block of stellate ganglia reduces neurotransmitter release and improves recovery following programmed ventricular pacing

**DOI:** 10.3389/fbioe.2026.1828505

**Published:** 2026-08-10

**Authors:** Shane A. Bender, Rami Aladham, Shyue-An Chan, Lily Defelice, Annie Kang, Kirellos Mikhail, Sahil Haridas, Jeffery L. Ardell, Kalyanam Shivkumar, Olujimi A. Ajijola, Corey B. Smith, Tina L. Vrabec

**Affiliations:** 1 Department of Physical Medicine & Rehabilitation, MetroHealth Medical Center, Cleveland, OH, United States; 2 Department of Physical Medicine & Rehabilitation, Case Western Reserve University School of Medicine, Cleveland, OH, United States; 3 Cardiac Arrhythmia Center David Geffen School of Medicine, University of California – Los Angeles (UCLA), Los Angeles, CA, United States; 4 UCLA Neurocardiology Research Program of Excellence, Los Angeles, CA, United States; 5 Department of Physiology and Biophysics, Case Western Reserve University, Cleveland, OH, United States; 6 Department of Biomedical Engineering, Case Western Reserve University, Cleveland, OH, United States

**Keywords:** arrhythmia, axonal modulation therapy, direct current nerve block, myocardial infarction, sympathetic efferent neurons, norepinephrine, fast-scanning cyclic voltammetry

## Abstract

**Introduction:**

The autonomic nervous system governs cardiac function through a finely-balanced interplay of sympathetic and parasympathetic activity. Following myocardial infarction chronic sympathetic hyperactivation destabilizes this balance, increases arrhythmia formation, and exacerbates cardiac dysfunction. Current treatments such as pharmaceuticals, surgical sympathectomies, and ablations, are either systemic, irreversible, or non-titratable, underscoring the need for targeted, adaptive interventions.

**Methods:**

Here we show that a closed-loop fuzzy logic controller, using real-time norepinephrine (NE) measurements via fast-scanning cyclic voltammetry as a feedback signal, can drive direct current (DC) electrical nerve block of the paravertebral sympathetic chain to achieve graded, on-demand reductions in cardiac NE release in both healthy (n = 6) and chronically infarcted (n = 5) male Yorkshire pigs.

**Results:**

Controller-directed block produced significant reductions in peak and sustained NE release at target setpoints of 33% and 66% in both animal models, with corresponding improvements in left ventricular systolic pressure recovery post-pacing.

**Discussion:**

These findings establish bioelectronic closed-loop sympathetic nerve block as a viable, reversible alternative to surgical sympathectomy. By delivering an electrical block to create an “on-demand sympathectomy”, refractory ventricular arrhythmia and post-infarction sympathetic dysregulation can be managed in real-time, side effects can be eliminated, providing the ultimate patient-specific treatment.

## 1 Introduction

The autonomic nervous system (ANS) provides comprehensive control of cardiac contractile function through a complex network of biological sensors and electrophysiological triggers referred to as the cardiac neuroaxis (Shivkumar and Ardell, 2016; Ardell et al., 2015). Cardiovascular status, reported by the sensors, can elicit neurological responses to maintain a healthy cardiac state. This adaptability serves the system well when an adverse event requires an adjustment to organ function in response to a change in the cardiac substrate, such as in myocardial infarction (MI). Unfortunately, the long-term effects of these adaptations, chiefly an increase in sympathetic neural activity, can leave the system in a less-than-optimal operating condition with elevated sympathetic tone contributing to long-term health issues. The use of neuromodulation therapies incorporating closed-loop control has the ability to respond to changing physiological conditions in real-time. In this study, neuromodulation is applied in response to elevated neurotransmitter levels in both normal and MI animals, to demonstrate the robustness and adaptability of this approach.

Current post-infarction treatment options include pharmaceuticals that act to reduce metabolic stress on the heart or to attenuate the effect of the elevated sympathetic tone (Exner et al., 1999; Larson et al., 2022; Zeppenfeld et al., 2022). However, pharmaceuticals act at the systemic level and produce side effects that may exacerbate co-morbidities and interact with other medications the patient might require(Ez Eddin et al., 2025; Butzner et al., 2022; Paolillo et al., 2021). Surgical interventions, such as stellectomy (Schwartz, 2014; Buckley et al., 2016b; Ajijola et al., 2012; Vaseghi et al., 2017) or renal ablation, have been evaluated as potential treatments that can specifically target sympathetic tone, but are irreversible and cannot be titrated to avoid unwanted side effects.

Electrical neuromodulation therapies provide targeted, on-demand interventions that can be rapidly turned on and off to provide a patient- and context-specific solution. There are several different possible neural targets that have been evaluated clinically. The most common form of electrical neuromodulation is stimulation, where electrical pulses increase the activity on a given nerve. Stimulation of the vagus nerve has emerged as a compelling target for cardiac neuromodulation due to its efferent projections into the heart and afferent control opportunities. Upregulation of parasympathetic activity to counteract elevated sympathetic tone has been applied clinically for arrhythmias (Stavrakis et al., 2017; Salavatian et al., 2016) as well as to preserve cardiac function in heart failure (Sabbah et al., 2011; Sabbah, 2011; Shinlapawittayatorn et al., 2014; De Ferrari, 2014; Beaumont et al., 2016). However, the large presence of afferent fibers (∼80%) in the vagus, as well as the diversity of the organs it innervates, presents challenges to deployment of vagal nerve stimulation (VNS) by generating a wide variety of off-target effects that must be addressed using proper parameter adjustments (Ardell et al., 2017).

Electrical nerve block (ENB) is a set of techniques that can disrupt action potential conduction, thereby lowering activity on a nerve. Like electrical stimulation, electrical nerve block can be applied at-will and is fast acting and reversible; it can also produce a graded effect by modulating the applied amplitude block to only block a subset of fibers in a nerve. The two main techniques used are kilohertz-frequency alternating current nerve block (KHFAC) and direct current nerve block (DC). Both have been used to modulate the paravertebral chain (Hadaya et al., 2022; Buckley et al., 2016a; Chui et al., 2017; Vrabec et al., 2024) to produce changes in cardiac output and to provide protection against ventricular fibrillation. KHFAC ENB uses 10 kHz–100 kHz charge-balanced waveforms to produce block; while KHFAC can be applied with standard platinum electrodes, it often produces “onset activity” when turned on, leading to a brief increase in activity on the target nerve. DC ENB, which is used in the present study, uses a continuous monopolar current to depolarize the nerve and keep it depolarized in an artificial refractory period. If DC is used with traditional electrodes, the current will produce harmful chemical species at the electrode interface that can cause damage to the nerve. This can be solved by using a special electrode known as the carbon separated-interface nerve electrode (CSINE) (Vrabec et al., 2019). This electrode is made of a high-capacitance suspension of carbon powder to prevent the formation of chemical reactants. The electrode is then connected to the nerve via a channel of conductive fluid (usually saline); this ionic connection further prevents any damage from occurring, as the electrode-electrolyte interface is not adjacent to the nerve as with traditional electrodes.

Electrical nerve block can be applied to any target that is currently treated with radio frequency (RF) ablation or surgical transection to provide a similar benefit while maintaining reversibility and mitigating off-target effects. Possible targets include renal RF ablation which has been applied for sympathetic downregulation but is typically used to control resistant hypertension (Wang et al., 2024; Sesa-Ashton et al., 2025; Biffi et al., 2023; Akinseye et al., 2021). The surgical transection of the cervicothoracic sympathetic chain has been shown to be an effective target for modulating sympathetic tone to reduce ventricular arrhythmias (Buckley et al., 2016b). This location has been evaluated as a potential target for the treatment of post-myocardial infarction ventricular arrhythmias resulting in a near complete reduction in ventricular tachyarrhythmias (Chui et al., 2017). This study builds on this work to implement this intervention in response to elevated norepinephrine (NE) levels.

Norepinephrine has been identified as potential biomarker for closed-loop control of sympathetic hyperactivity due to its release from post-ganglionic sympathetic efferents, particularly during heart failure (Cohn et al., 1984; Jänig, 2006). However, existing methods for the measurement of NE are lacking, making it difficult to evaluate this important biomarker in a routine clinical setting. Current technologies cannot identify NE levels in real-time, instead depending on the collection of interstitial fluids via microdialysis (Ardell and Armour, 2016) and laboratory measurements to evaluate samples. A novel electrochemical measurement technique, fast scanning cyclic voltammetry (FSCV) has successfully been used to measure NE in real-time in porcine experiments (Chan et al., 2020; Vrabec et al., 2024). In FSCV, a fine platinum wire is inserted into the myocardium of the ventricle wall and a sawtooth command voltage waveform is applied to the electrode, resulting in oxidation of catecholamines to produce quinones and reduction of quinones to convert back to catecholamines. Charge current is measured and the amplitude at the oxidation potential for NE is measured to provide a scaled index of NE at the surface of the electrode. This waveform is repeated throughout the measurement duration to determine the level of NE in real-time. Neuropeptide Y (NPY) is an additional potential biomarker for long-term changes in cardiac diseases (Ajijola et al., 2015; Chow et al., 2017; Gullestad et al., 1998; Kalla et al., 2020; Ullman et al., 1994). NPY can be measured in real-time in a similar way to NE using a capacitive immunoprobe (CI). The electrode is functionalized at the measurement surface with an antibody against a target antigen (NPY). The binding of the antigen to the electrode surface increases the capacitance of the system and provides a measurement of NPY at the electrode tip (Kluge et al., 2021). This technology has been deployed in porcine experiments and compared to NE release under cardiac stressors both with and without a neuromodulatory intervention (Kluge et al., 2021; Vrabec et al., 2024).

Closed-loop electrical neuromodulation involves altering the electrical signals delivered to a nerve in response to a change in a patient’s condition (Zanos, 2019). Whereas traditional neuromodulation devices give a predetermined prescribed stimulation pattern regardless of patient state or disease condition, closed-loop devices are able to respond to measured inputs, such as patient activity or biomarkers that indicate disease state. Closed loop neuromodulation has been used clinically in spinal cord stimulation for pain relief (Russo et al., 2018; Russo et al., 2020; Mekhail et al., 2020), deep brain stimulation for Parkinson’s Disease (Wang et al., 2023) and epilepsy (Geller et al., 2017), genital nerve stimulation for bladder control (Karam et al., 2016), and vagus nerve stimulation for cognitive recovery after spinal cord injury (Kilgard et al., 2025), among others. For the treatment of cardiovascular disease, the most popular option is vagus nerve stimulation (VNS). Closed-loop vagus nerve stimulators show promise in treating not only cardiac disease, but also in other vagus-mediated diseases such as depression, pain, epilepsy, and obesity (Mathews et al., 2025; Ottaviani et al., 2022; Bender et al., 2024). Another active area of study is the stimulation of carotid baroreceptors (de Leeuw et al., 2017; Seravalle and Grassi, 2015) or nerves of the sympathetic trunk (Hosokawa and Sunagawa, 2016) for control of blood pressure. In this study, instead of stimulating a nerve to increase its activity, we are using closed-loop neuromodulation to electrically block sympathetic inputs to the heart.

Conventional controllers such as proportional/integral/derivative (PID) controllers often have difficulty providing robust control under physiological conditions so a novel control scheme based around a fuzzy logic controller (FLC) was selected for this study. FLCs are good at generating a continuous control function from a discrete set of rules; as such, it is good for translating plain-language observations into a control scheme in the absence of a complete mathematical model of the system. Previous work from our group has shown success with using this technique for controlling vagus nerve stimulation and block for control of heart rate (Bender et al., 2025; Bender et al., 2021; Bender et al., 2024).

MI produces profound changes in cardiac tissue causing heterogeneities that facilitate reentrant pathways that result in arrhythmias. Although in this study, the controller only incorporated the input from one probe outside of the scar zone, probes were placed both in the scar as well as in adjacent tissue. Previous studies have demonstrated that the release of NE and NPY exhibits a different profile in the scar verses the unaffected region of the heart (Vrabec et al., 2024). This heterogeneity is a potential indicator of the magnitude of arrythmia and the expected outcome as a result of cardiac stressors such as programmed pacing. Programmed ventricular pacing has been leveraged in this study as a repeatable method for eliciting a strong sympathetic response. Compared to the traditional MI therapies of medications, implantable pacemakers and defibrillators, and sympathectomy, the use of closed-loop electrical neuromodulation has the potential to provide several key advantages. The use of electrical nerve block on the sympathetic chain provides an “on-demand sympathectomy”, aiming to provide the efficacy of sympathectomy with the rapid application and reversibility of electrical neuromodulation. As an advantage of being an electrical neuromodulation therapy, the effect of the therapy occurs nearly instantly and can be adjusted quickly without an extended washout period, like what is required when adjusting medication. In fact, by closing the loop, the degree of therapy can be modulated fast enough for real-time titration without the intervention of the patient or physician. This is an improvement over open-loop therapies like medications, or more recently open-loop vagus nerve stimulation.

In this study normal and MI pigs undergo programmed pacing to determine the efficacy of closed loop neuromodulation to downregulate sympathetic hyperactivity. Measurements of NE in unaffected cardiac tissue were used to determine operational levels for the controller. NE and NPY measurements were also collected in additional regions of the heart including the border zone in MI animals. These measurements both show the efficacy of closed loop neuromodulation as well as providing insights into mechanisms and potential targets for future interventions.

## 2 Methods

### 2.1 Ethical approval and study design

All animal experiments were approved under University of California–Los Angeles (UCLA) Institutional Animal Care and Use Committee (IACUC); protocol 2016–084. Experiments were performed in accordance with the *National Institutes of Health’s Guide for the Care and Use of Laboratory Animals.*

A combination of 5 chronically infarcted and 6 healthy control male Yorkshire pigs (*Sus scrofa*, 50–60 kg) were used in these experiments.

The chronic and terminal experiements are similar to what we have described previously (Vrabec et al., 2024). A more detailed description of the chronic and terminal surgical methods can be found in the Supplementary Material. Breifly, in chronically infarcted animals, the left anterior descending coronary artery was embolized with polystyrene microspheres to occude the vessel and lead to an infart. Infarcted animals were recovered for 6 weeks before the terminal procedure. Following the 6 weeks of recovery post-infarct, a scar is visible on the anterio-lateral aspect of the left venticle. Scar size was measured with intracardiac echocardiography, described below.

**FIGURE 1:**
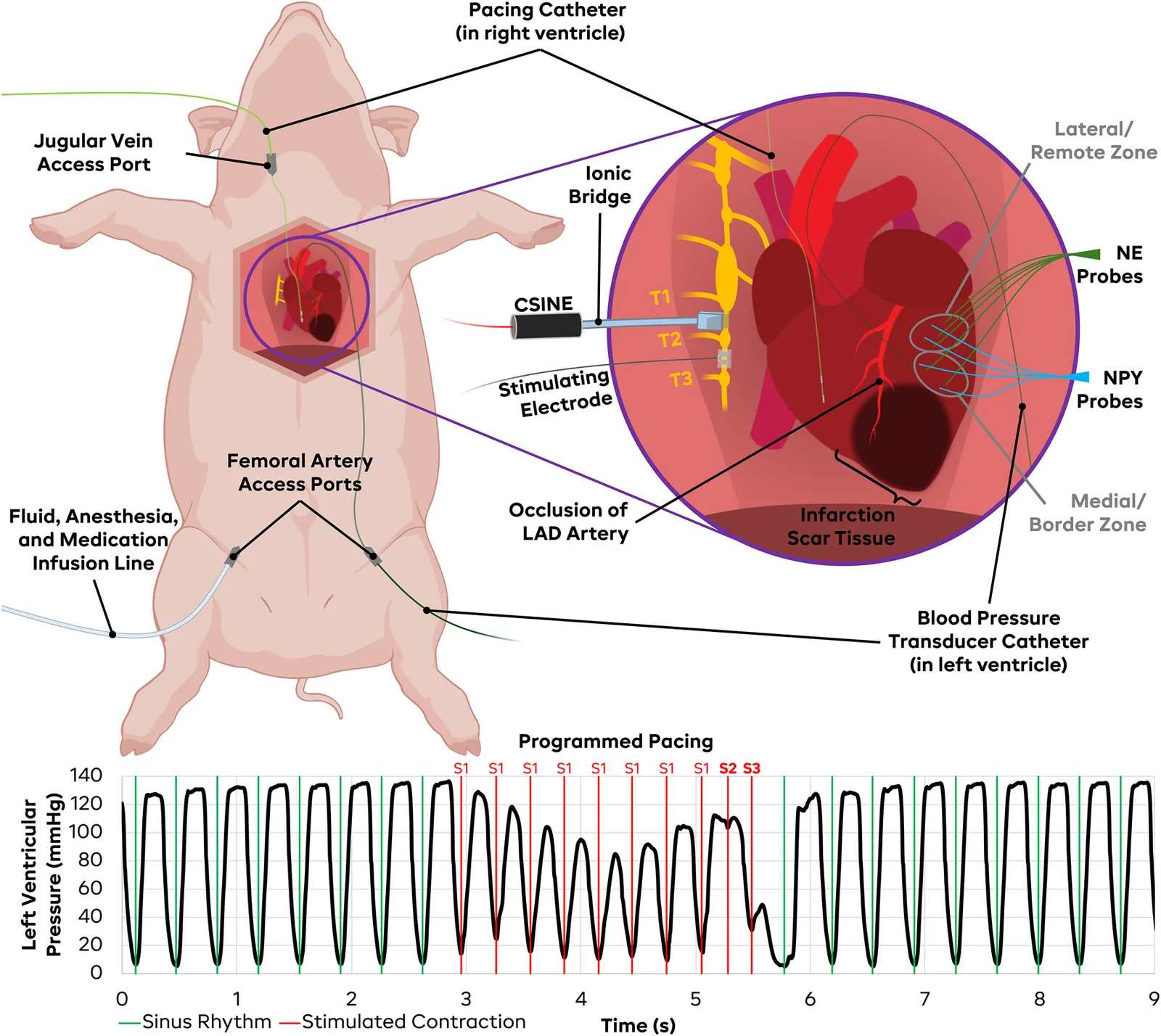
The surgical preparation and pacing protocol for the terminal experiments. The top portion of the plot shows the surgical setup and instrumentation for these experiments. For chronically infarcted animals, the site of the left anterior descending (LAD) artery occlusion is labeled, along with the resulting scar tissue. The bottom portion shows an example of the decremental programmed pacing protocol used in these experiments.

The terminal procedure for both the healthy and chronically infarcted animals can be seen in Figure 1. The femoral arteries were accessed bilaterally for blood pressure catheter insertion and fluid delivery. The right jugular vein was accessed for insertion of the ventricular pacing catheter. A sternotomy was performed to expose the heart and paravertebral chain. In the inset, a CSINE (Vrabec et al., 2019) for blocking current delivery was attached to the chain at the T1-T2 level, and a bipolar platinum stimulating electrode (for assessing block threshold) was placed at T2-T3. The heart had a pacing catheter placed in the right ventricle, and a pressure-measuring catheter placed in the left ventricle. The placement of the 4 to 5 fast-scanning cyclic voltammetry (FSCV) probes and the 4 capacitive immunoprobes (CI) are shown. The NE electrode with the most pronounced response was used to feed into the controller, while the other NE electrodes and the NPY electrodes were used to collect ancillary global data.

### 2.2 Experimental protocol

#### 2.2.1 Echocardiography

In a subset of the MI animals, intracardiac echocardiography was used to measure left ventricle (LV) internal dimensions in systole (LVIDs) and diastole (LVIDd), and calculate LV ejection fraction (LVEF) using the monoplane method. Equivalently gathered data from historical healthy controls was used for direct comparison.

#### 2.2.2 Block thresholding

A more in-depth description of the methods to find the block threshold can be found in the Supplementary Material. In brief, stimulation was applied to the right sympathetic chain at T2-T3 to elicit an increase in heart rate. Direct current electrical nerve block was applied at the T1-T2 level of the chain to counteract the effect from stimulation. Block was applied for 2 min prior to stimulation, and then left on during a following 20 s of sympathetic stimulation. The minimum current needed to completely attenuate any change in heart rate from the stimulation was termed the “block threshold”, and was used to normalize the applied blocking current between animals and sets.

#### 2.2.3 Trial format

All trials followed the format: 2 min of baseline measurements, then 5 min of programmed pacing (and block if applicable, then at least 2 more minutes to record the recovery from programmed pacing. The pacing protocol is described below.

Trials were organized into sets of 3. An initial unblocked trial was run first to measure the expected change in norepinephrine (NE) concentration during the programmed pacing using fast-scanning cyclic voltammetry, described previously (Vrabec et al., 2024). The normalization “100%” value was selected as the highest sustained current in the last 2 minutes of the programmed pacing, rounded to the nearest 5 nA (nA). For these experiments, peak values of 75 nA–300 nA were seen. Following the unblocked trials, 2 trials were run with a closed-loop, fuzzy logic controller administering direct current nerve block to reduce the NE release by 33% or 66%; these percentages were run in a randomized order for each set. A more detailed description of the measurement and controller techniques can be found in the Supplementary Material.

#### 2.2.4 Electrochemical detection

More in-depth methodologies for the electrochemical sensing techniques used can be found in the Supplementary Material. In brief, the FSCV signals were acquired by applying a sawtooth waveform to a platinum wire electrode between −0.5 and +1.2 V vs. a stainless steel reference electrode (hypodermic needle). The stainless steel reference electrode, as well as changes in electrode surfaces due to biofouling in tissue (Mughrabi et al., 2023), may cause shifts in the reduction and oxidation potentials of norepinephrine, but as this is the only electrically active catecholamine expected to be released in these experiments, and the measurement potential was set for each animal and set individually, this was considered acceptable. Changes in current from the oxidation of norepinephrine were recorded, and are reported in absolute levels (nA) or normalized changes (%). Selectivity of these measurements was confirmed in prior *in vivo* and *in vitro* experiments; these experiments are summarized in the Supplementary Material.

CI measurements were acquired by applying an asymmetric, discontinuous square wave, consisting of 20 ms each at the following potentials, in order: 0 mV, 100 mV, 0 mV, −5 mV to a platinum wire electrode vs. a silver/silver chloride (Ag/AgCl) reference electrode. An increase in the amount of NPY present will result in an increase in capacitance at the probe surface. The capacitive measurement occurs on the transition from 0 to 100 mV. The −5 mV step is designed to repel the previously bound NPY and prepare the probe for the next measurement.

#### 2.2.5 Pacing protocol

The perturbation to elicit NE and NPY release was right ventricular decremental programmed pacing, acting as a sympatho-excitatory reflex stressor. The goals for pacing did not include induction of ventricular tachycardia (VT) or ventricular fibrillation (VF), but this did occasionally occur. Previous work has shown that blocking sympathetic input to the heart decrease VF inducibility significantly (Chui et al., 2017), but that was not the focus of the present study. If VT or VF occurred and the subject required defibrillation, the experimental set was abandoned, an extra 45-min wait was added, and a new set was restarted.

The programmed pacing (PP) was composed of stimulus train of 8 pulses (S1), followed by a series of up to 2 extra-pulses (S2-S3), followed by a 3-s wait. The S1 pulses were given at an interval 20% shorter than the resting cycle length prior to pacing. Initially, only one extra-pulse (S2) is administered, starting at an interval of 400 ms (or the S1 interval if that were to be smaller). For each successive pulse train, the S2 interval was lowered by 20 ms, until the S2 stimulus failed to produce a contraction (unsuccessful capture) or until an interval of 200 ms was reached. If a given pulse interval failed to produce a contraction, it was labeled as the effective refractory period (ERP). After the S2 ERP was found, the S2 was stimulus administered at the ERP +20 ms (to ensure successful capture). The S3 stimulus was added following S2, starting at the S2 interval +30 ms, and decreasing until the S3 failed to illicit a contraction, or 200 ms was reached. Once both ERPs had been found, the S3 was run at 20 ms longer than ERP to ensure successful capture, the full S1-S2-S3 train was repeated until a duration of 5 min of pacing had elapsed. An example of the pacing protocol can be seen at the bottom of Figure 1.

### 2.3 Statistics

For comparative metrics, Wilcoxon Signed-Rank tests were performed between groups of data. The Wilcoxon test was chosen due to the non-normality of the measured data, in part due to a small number of repeats for each category, and in part due to the inherent variability in the catecholamine measurements. In analyses of variances, a superiority test for the ratio of standard deviations was performed. All statistics were run in JMP v18 (JMP Statistical Discovery, Cary, NC, USA).

## 3 Results

### 3.1 Echo infarct sizes

Echocardiography was used to assess functional impairment of infarcted hearts, and revealed that MI animals had significantly weakened left ventricular (LV) function and structure compared to healthy controls, including a lower left ventricular ejection fraction (LVEF) (51.49% ± 11.0% vs. 65.9% ± 5.0%, P < 0.03), larger left ventricular internal diameter at end-diastole (LVIDd) (3.35 ± 0.92 cm vs. 2.28 ± 0.44 cm, P < 0.04), and at end-systole (left ventricular internal diameter at end-systole, LVIDs; 2.97 ± 0.72 cm vs. 1.12 ± 0.29 cm, P < 0.001). The anterior wall was thinner in diastole (LV anterior wall thickness; 0.41 ± 0.02 cm vs. 0.49 ± 0.02 cm, P < 0.001), but not significantly different in systole.

### 3.2 NE release measured by the controller

#### 3.2.1 Trial data

**FIGURE 2:**
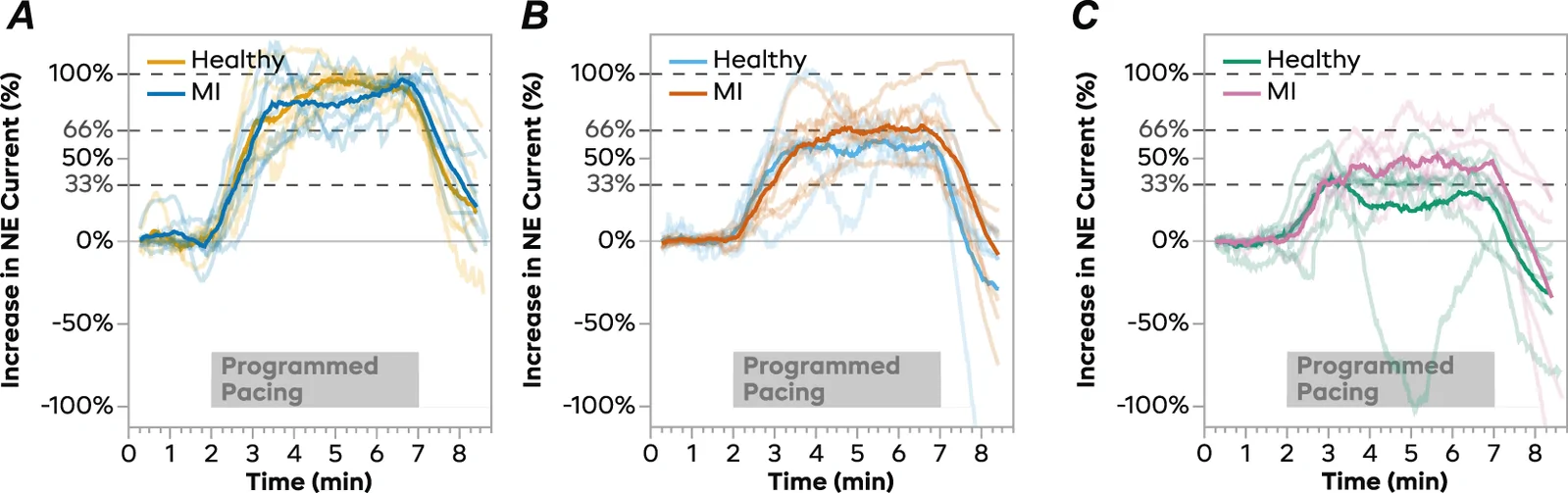
The above plots show the normalized NE current traces for each trial in both healthy and MI animals. The bolded traces represent the mean value at each timepoint. From left to right, there is the unblocked baseline trials **(A)** the 33% reduction controlled trials **(B)** and the 66% reduction control trials **(C)** In general, the current trajectories appear similar between the healthy and MI animals, aside from a single 66% reduction trial in a healthy animal, where the norepinephrine spontaneously plunged below the baseline soon after block was applied.

In both the healthy and infarcted animals, the controller was able to recognize when the norepinephrine had reached the setpoint and turn the blocking current up to mitigate it. Figure 2 shows each data trial for the unblocked and blocking trials. At a high-level, the controller is able to mitigate the NE release to approximately the setpoint value. More formal tests of this are described below. The controller is active throughout the duration of the programmed pacing in the 33% and 66% reduction trials.

#### 3.2.2 Peak NE current

**FIGURE 3:**
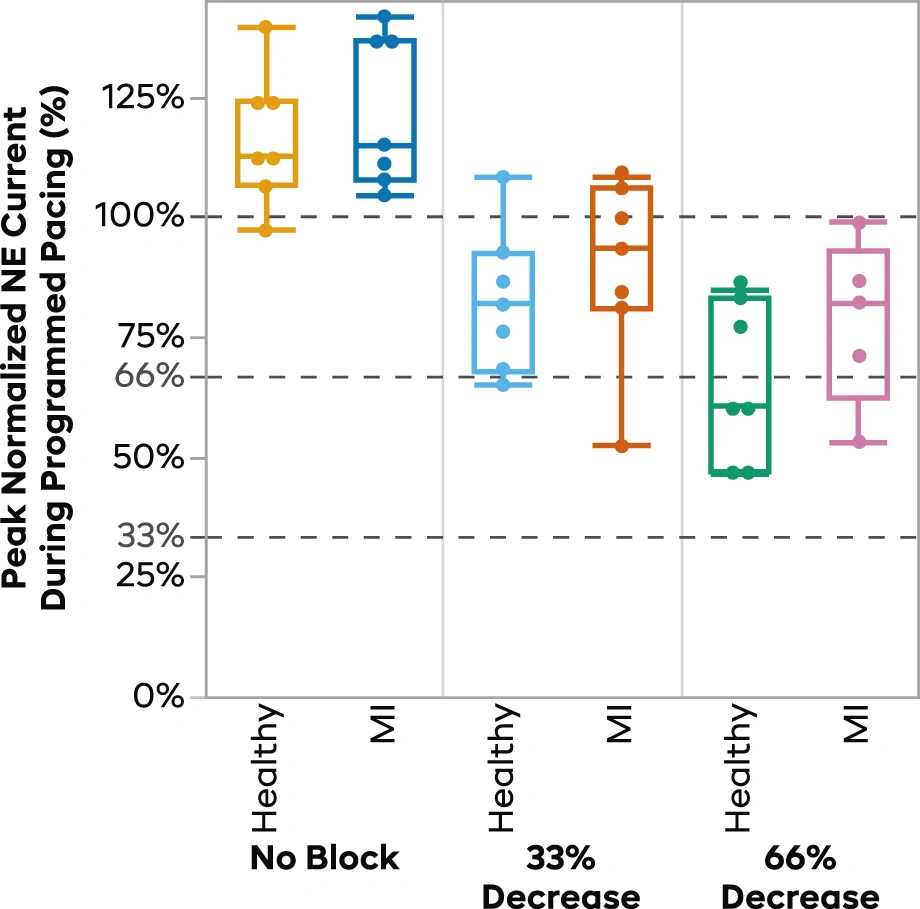
This plot shows the peak normalized current for each type of trial. For the healthy animals, the unblocked trials had the highest peaks, (median 112.5%), the 33% reduction trials were lower (median 81.8%) and the 66% reduction trials were the lowest (median 60.6%). The MI pigs followed the same order, with the unblocked trials having the largest peaks, (median 115.2%), the 33% reduction trials were lower (median 93.5%) and the 66% reduction trials were the lowest (median 82.3%).

The peak normalized NE current during programmed pacing was analyzed for each of the trials and plotted in Figure 3. Significant differences were seen in both animal models between the No Block and 33% Reduction trials (Healthy p = 0.0049; MI p = 0.0073) and between the No Block and 66% Reduction trials (Healthy p = 0.0022; MI p = 0.0058), but no significant difference was seen between the 33% and 66% reduction trials (Healthy p = 0.0967; MI p = 0.3299). No significant differences were seen between the healthy and MI animals at any blocking setpoint (No Block p = 0.7015; 33% Reduction p = 0.3711; 66% Reduction p = 0.2556).

#### 3.2.3 Mean and settling NE current

**FIGURE 4:**
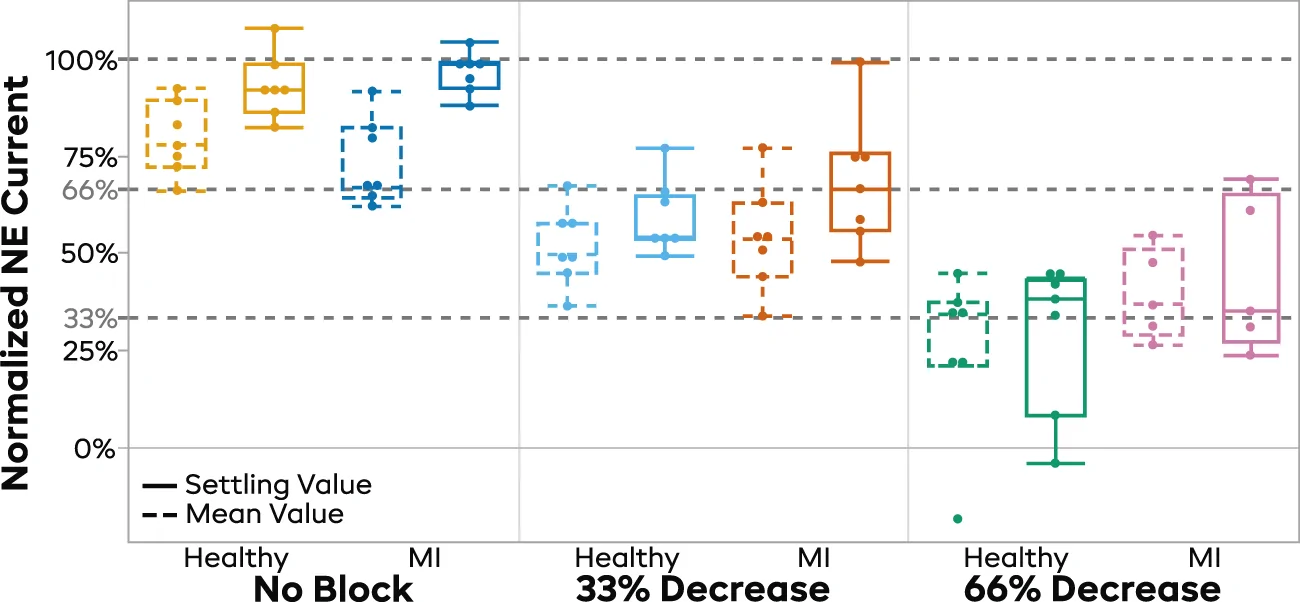
The dashed box plots show the mean normalized current during the programmed pacing for each type of trial. In both healthy and MI animals, an increase in block lowered the mean NE currents measured. The solid box blots show the settling value for each type of trial, represented by the mean of the last 30 s of pacing. Similar to the mean values, an increase in block lowered the mean NE currents measured in both healthy and MI animals.

Figure 4 shows both the mean normalized NE current (dashed) and the settling value (solid). The mean was the average current during the entirety of the programmed pacing, and the settling value was the average for the last 30s of programmed pacing.

The mean currents during pacing were typically lower than the setpoint due to the ramp up period at the beginning of the trial. There was not a significant difference between means of healthy or MI animals (No Block p = 0.3067; 33% Reduction p = 0.8983; 66% Reduction p = 0.2556). There were differences in mean currents seen between the block setpoints, as would be expected. For the healthy animals, there were significant differences between setpoints (No Block/33% Reduction p = 0.0033; No Block/66% Reduction p = 0.0022; 33%/66% Reductions p = 0.0049). For the MI Animals, there were significant differences between the unblocked trials and the blocked trials (No Block/33% Reduction p = 0.0152; No Block/66% Reduction p = 0.0058), but no difference was seen between the two blocked trials (33%/66% Reductions p = 0.1044).

The settling values tell a similar story to the means, as there was also not a significant difference between healthy and MI animals (No Block p = 0.2502; 33% Reduction p = 0.3067; 66% Reduction p = 0.6261). Also similarly to the means, there were significant differences in settling values between all setpoints for the healthy animals (No Block/33% Reduction p = 0.0022; No Block/66% Reduction p = 0.0022; 33%/66% Reductions p = 0.0022), while the MI Animals showed significant differences between the unblocked trials and the blocked trials but not between the two blocked trials (No Block/33% Reduction p = 0.0215; No Block/66% Reduction p = 0.0058; 33%/66% Reductions p = 0.1044).

### 3.3 Ancillary NE and NPY data

Broadly speaking, there was little difference seen between the blocked and unblocked trials in either the non-controller NE or NPY measurements. This can in part be attributed to smaller signals captured by the other electrodes combined with high inter-electrode variability. A more robust analysis of these measurements can be seen in the Supplementary Material.

### 3.4 Blocking currents

**FIGURE 5:**
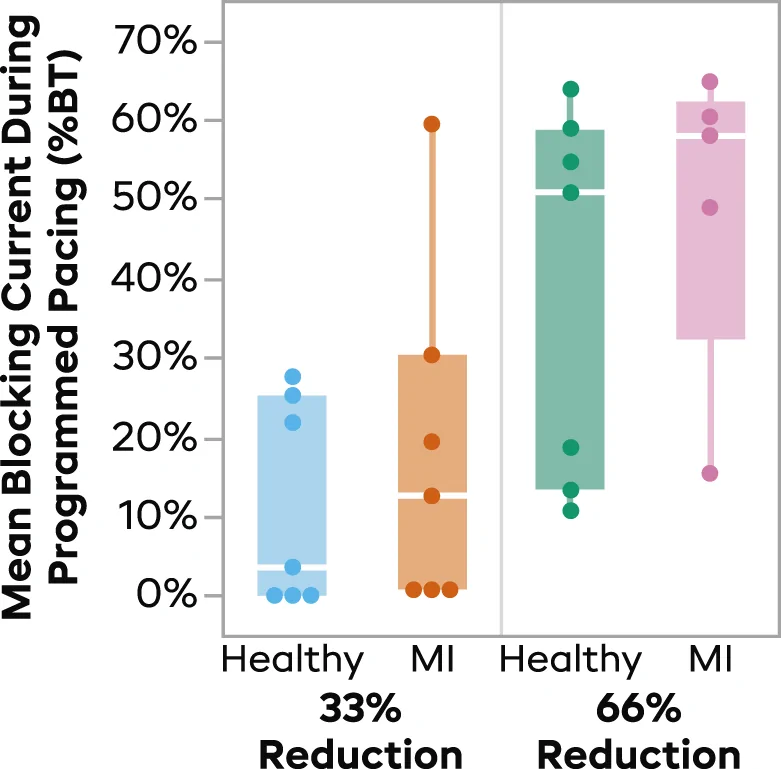
The above plot shows the mean blocking current delivered during the programmed pacing for both the healthy and MI animals, reported as a function of the block threshold (%BT). For both cases, the 66% reduction trials saw more blocking current than the 33% reduction trials, but the differences were not statistically significant.

The amount of block delivered was controlled by the fuzzy logic controller, and the average amount of block (reported as a fraction of the block threshold) is seen in Figure 5. No significant differences were seen between MI and healthy animals in either the 33% reduction trials (p = 0.7015) or the 66% reduction trials (p = 0.4168). Strong but non-significant differences were seen between the two setpoints in both the healthy (p = 0.0553) and the MI animals (p = 0.0513); when the two animal models were combined, a significant difference was seen between the 33% and 66% reduction trials (p = 0.0059). One trial in healthy animals and three in the MI animals required very little block (<0.5% BT average) to achieve a 33% reduction.

### 3.5 Cardiac functional data

#### 3.5.1 Left ventricular pressure

**FIGURE 6:**
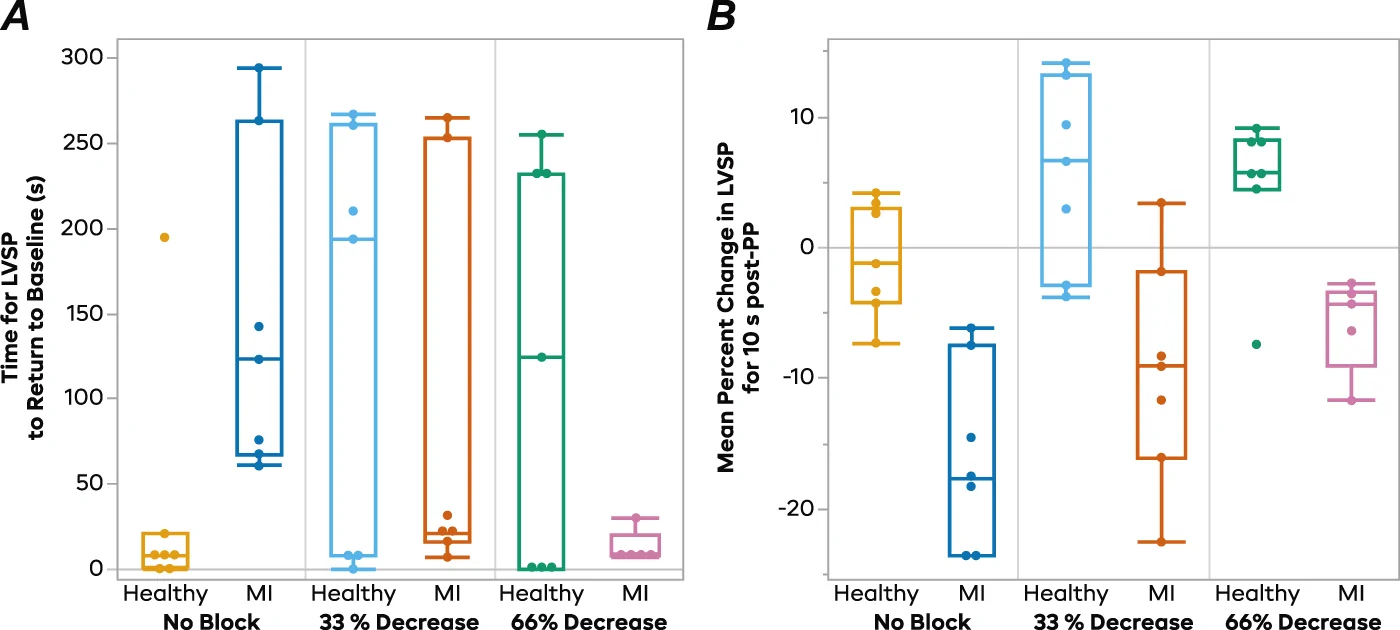
The left plot **(A)** shows the change in left ventricular systolic pressure after pacing compared to the pre-pacing baseline. Significant differences were seen between MI and healthy animals at each setpoint, with the MI animals showing significant decreases in pressure following pacing, where healthy animals showed increases. There were also significant differences between the unblocked and 66% reduction trials in both MI and healthy animals. The right plot **(B)** shows the time after pacing where the LVSP returned to the baseline level. Responses were quite varied for most groups, but in MI animals the 66% decrease trials recovered significantly faster than the unblocked trials.

The left plot on Figure 6A shows the change in left ventricular systolic pressure (LVSP) as a result of programmed pacing compared to the pre-pacing baseline, as represented by the mean systolic pressure for the 10 s following pacing. Significant differences were seen between healthy and MI animals at each setpoint (no block p = 0.0033; 33% reduction p = 0.0152; 66% reduction p = 0.0345). Between setpoints, significant differences were seen between the unblocked and 66% reduction trials for both healthy (p = 0.0298) and MI (p = 0.0230) animals. No other significant differences were seen between setpoints in healthy (unblocked/33% reduction p = 0.0967; 33%/66% reductions p = 0.7015) or MI (unblocked/33% reduction p = 0.2013; 33%/66% reductions p = 0.5160) animals.

The right plot on Figure 6B shows the time following programmed pacing where the LVSP recovered to the pre-pacing baseline level. Significant differences were seen between the healthy and MI animals for the unblocked trials (p = 0.1510), but not for the blocked trials (33% reduction p = 1.000; 66% reduction p = 0.7445). Between setpoints, no differences were seen in healthy animals (unblocked/33% reduction p = 0.4047; unblocked/66% reduction p = 0.5201; 33%/66% reductions p = 0.6069). In MI animals, a significant difference was seen between the unblocked and 66% reduction trials (p = 0.0058), but not between the other comparisons (unblocked/33% reduction p = 0.0967; 33%/66% reduction p = 0.1044).

#### 3.5.2 Heart rate

**FIGURE 7:**
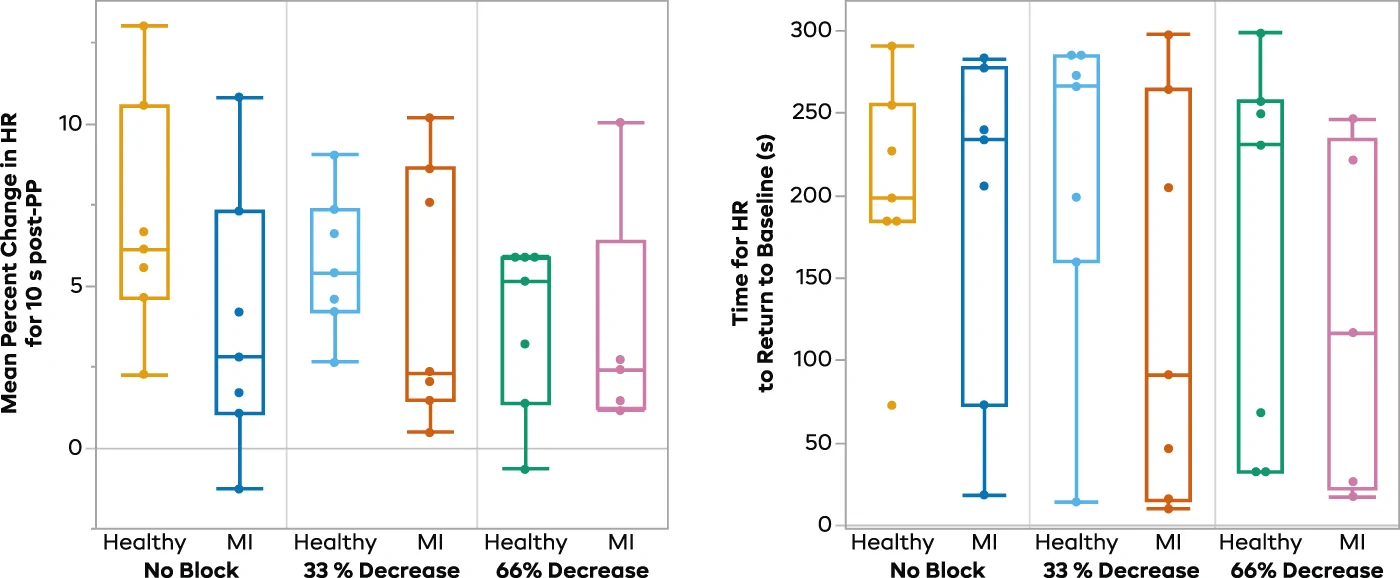
The left plot **(A)** shows the change in heart rate after pacing compared to the pre-pacing baseline. In general, the MI animals saw marginally smaller increases in heart rate after pacing, but no significant differences between any groups were seen. The right plot **(B)** shows the time after pacing where the heart rate returned to the baseline level. Responses were quite varied, and no significant differences were seen between any groups.

The left plot on Figure 7A shows the change in heart rate (HR) as a result of programmed pacing compared to the pre-pacing baseline, as represented by the mean heart rate for the 10 s following pacing. Hemodynamic measurements could not be taken during the pacing, as the pacing itself masks what the unperturbed state of the heart would be. Between each combination of pairs between healthy/MI animals and controller setpoint, no significant differences were seen. In general, responses were quite varied, and although it appears the MI animals may have slightly smaller increases in HR following pacing, there are not sufficient data points to reach any statistically significant conclusion.

The right plot on Figure 7B shows the time following programmed pacing where the heart rate recovered to the pre-pacing baseline level. No significant differences were seen between any combination of pairs between healthy/MI animals and controller setpoint. Recovery times were also quite varied, ranging from <30 s to 5 min.

## 4 Discussion

### 4.1 NE release in the controlled electrode

In general, the closed-loop control scheme used in these experiments was able to execute fairly robust control over the norepinephrine content at measured electrode. The average current during pacing and the settling value (Figure 4) both show significant decreases in NE release between the unblocked and blocked trials. The healthy control animals saw a somewhat stronger level of control, with the 66% reduction being significantly lower than the 33% reduction in both the mean and settling values. The MI animals did not see quite as robust control; while both NE reduction setpoints differed from the unblocked trials, the 33% and 66% reductions were not seen to be significantly different. This can be attributed to a larger spread being seen in the 66% reduction trials in the MI animals, where for some animals, unilateral block did not appear to create the desired reduction in NE release. This can also be seen in raw traces in Figure 2, where the MI animal average remains above the 33% NE level, while the rest of the average traces stay under their respective setpoints. This may indicate a limitation for future clinical applications, where if NE release mitigation below ∼50% is desired in patients who have suffered a heart attack, bilateral paravertebral chain block may be required. This was also the main difference between the healthy and MI animals, as the robust control appeared to be achieved in both the 33% and 66% reduction trials in the healthy animals, but the 66% reduction could not quite reach the desired setpoint.

Similar conclusions can be drawn when analyzing the peak NE current. While there are significant reductions in NE between the unblocked and both blocked trials, there was no difference between the two blocked trials in either healthy or MI animals. Part of this may be due to controller design, which only turned the blocking current on once the setpoint had been reached. In another implementation, the block may be turned on sooner based on a lower threshold, by analyzing the slope of the NE current change, or by incorporating other inputs into the controller (e.g., heart rate, activation recovery interval, etc.). It remains to be seen if a large, short burst in NE release or a sustained lower increase is more arrhythmogenic; if the former is worse, controller adjustments would need to be made to increase the response time of the controller to better attenuate the sympathetic response. If the duration of a smaller NE increase proves to be a more clinically relevant metric, the implemented control scheme may be effective.

Despite a very complex setup and a diverse set of biological and physiological conditions, the programmed pacing protocol used for these experiments was able to generate a robust and consistent response once the normalization was performed. While normalizing NE currents may not be immediately clinically relevant, for the goals of these experiments, it provided a way to compare results of the control scheme across a spectrum of animals, both healthy and with various sizes of infarction scars.

### 4.2 Blocking currents required to achieve setpoints

The amount of block delivered to achieve each setpoint followed the expected pattern, where more reduction in NE release required more block. A few trials required very little block, which may indicate that the NE release for these trials happened to be lower than baseline, or that the stellate was very responsive to being blocked for a short period of time. The amount of blocking current delivered was limited to 150% of the block threshold (BT_120_, 120 s block threhsold); increasing this maximum value may allow for a faster response to detected increases in NE but may also lead to controller instability. High blocking currents could lead to interference in the measurement of the catecholamines; as such, care was taken to ensure that the blocking current did not cause interference during each set of these experiments. Interference was tested by applying a brief (<5 s) pulse of 150% BT_120_ between trials; interference would show up as a sharp step in measured NE current on the onset and offset of the pulse. Moving the blocking return electrode closer to the nerve significantly reduces any potential for interference between the FSCV and block equipment.

### 4.3 Local vs. global NE control

Outside of the controller, the level of control in NE release falls off greatly. There were no significant differences seen between any of the setpoints in any of the zones, in neither the healthy nor MI animals. For the basal measurements, a lack of difference is expected, indicating that each trial started in a similar state. For mean values, this may be explained by overall low NE release measured at the non-controller electrodes, but there were also no differences seen in the peak release. This variability could be due to the high degree of variability in these measurements, disparate innervation from the left and right paravertebral chains, or simply that a single mode of control does not achieve global changes in NE release. It is also unknown how much variability is caused by the proximity of the probe to a sympathetic fiber, and whether this proximity is required to achieve a useful measurement signal. Some changes were seen between the healthy and MI animals, specifically in the peak currents, but in general the lack of correlation to the setpoint did not help elucidate any changes that may occur with chronic infarctions.

### 4.4 Effect on NPY

The insights gained from the NPY recordings are clouded due to the overall low NPY release caused by our pacing protocol. The pacing protocol used in these experiments was quite strenuous and induced ventricular fibrillation 18 times over the 20 animals used, so it is unclear why NPY release was not greater. It is possible that the animals that fibrillated had higher NPY levels, but due to the fibrillation we could not finish an experiment set; this would induce a sampling bias towards low-NPY-release animals.

While the amplitudes of the NPY release did not show any significance, the spread of the NPY releases did decrease between the unblocked and blocked trials. This may indicate that, in responsive animals, NPY release was attenuated as a result of paravertebral chain block. Regardless of the cause, reducing heterogeneity in catecholamine release should provide an anti-arrhythmic effect, especially post-infarction.

### 4.5 Functional effects

#### 4.5.1 Left ventricular pressure

Unlike heart rate, significant changes in response to the level of block were seen in left ventricular systolic pressure following programmed pacing. Differences between healthy and MI animals can be partially attributed to the noncontractile scar tissue as a result of the infarction. However, there were differences between setpoints in the same animal models as well; specifically, between the unblocked trials and the 66% NE reduction trials in both healthy and MI animals. In both cases, the pressure increased as the amount of NE decreased, with the healthy animals going from a small increase after pacing to a larger one with increased block, while the MI animals had from a substantial pressure decrease that was reduced as the NE release was diminished. This seems contradictory, as a decrease in sympathetic input is typically associated with a decrease in contractility and pressure. One explanation may be that the decrease in NE lowered a reflexive parasympathetic response, which would then lead to an increase in contractility. Another may be that left-side sympathetic activity increased as a result of right-side block (Fioretto et al., 2011). A third cause could be that the blocking of afferent activity on the paravertebral chain altered the central reflex control; this was also seen during the block thresholding trials, where the heart rate and blood pressure would increase and then level off when the blocking current was first turned on.

The recovery times for LVSP to return to baseline were largely bimodal, with clusters under 60 s and above 180s with few times in between. This bimodal distribution was not seen in the unblocked trials in MI animals, which had generally longer recovery times (all >60 s). In MI animals, a 66% reduction in NE was associated with a significantly decreased recovery time, while nonsignificant differences were seen between the other groups. This could indicate that the sympathetic block prevented the neurochemical concentration changes that normally occur from programmed pacing, leading to a reduced deviation from the baseline, leading to faster recovery times.

#### 4.5.2 Heart rate

No significant trend was seen in the change in heart rate as a result of pacing. The responses seen were quite varied, although most trials saw less than a 10% increase in heart rate following pacing, with 2 even seeing a decrease. A similar lack of significant differences was seen in time post-pacing to return to the baseline heart rate. Both of these could be partially explained by our not controlling, nor even measuring, atrial catecholamine release. Since pace-making is typically at the sinoatrial node in the right atrium, poor control over this region may have been seen with unilateral stellate block, or that catecholamine release in the atria does not exactly parallel release in the ventricles. Seeing the wide range of measured NE current in the ventricles, it follows that the differences between the ventricles and the atria may be even more varied.

### 4.6 Limitations

Measurements from probes other than the one used as the controller input show that global control of catecholamines may not be as precise as control over a local area of interest. Despite changes seen in functional measurements of the heart (namely, LVSP), it is unclear to what degree the unilateral application of the block affects the ability for global control. Future studies using bilateral paravertebral chain nerve block may provide more control over global catecholamine release than unilateral block; however, bilateral block may be clinically undesirable, as this would be more intrusive to the patient in a procedure that would already be somewhat invasive.

Although there are well-established sex-related differences in both electrophysiological measurements and the underlying physiology itself (Linde et al., 2018), this study does not investigate these differences. Future studies with larger numbers of animals will be needed to determine the extent of the sex-based differences in efficacy of this therapy.

This study was meant to demonstrate that real-time, closed-loop reduction of norepinephrine release with electrical nerve block is possible, compared to the proactive block that has been demonstrated previously. This study did not determine what the best therapy for a given condition might be. It is possible that a maximal reduction in NE release may provide the most benefit to a patient. In that case, applying a constant, maximum block when an increase in NE is detected may be more beneficial than a tighter degree of control. Further, long-term studies are needed to determine what the optimal level of block is.

While long-term kilohertz-frequency alternating current (KHFAC) nerve block has been applied chronically (Kapural et al., 2024), the effects of long-term direct current application are less-known. Proper electrode design can be used to avoid any chemical reactant formation to prevent damage, and recent work has shown no detrimental effects to nerve fibers and support cells to long-term direct current (Fernandez Brillet et al., 2026). What has not been studied are the potential systemic adaptations made by the neurocardiac system caused by intermittent attenuation of sympathetic tone.

In addition to the unilateral block provided, the use of anesthetized animals provides another potentially confounding factor. It is well-known that anesthesia can blunt cardiac reflexes, and while alpha-chloralose was used to leave these reflexes mostly undisturbed, the degree to which the autonomic feedback control is affected is still unknown.

### 4.7 Translation and future targets

Pacemakers and implantable cardioverter-defibrillators are closed-loop devices that can detect arrhythmias and apply stimulation to correct them; unfortunately, by nature of their direct stimulation of the heart, they are unable to prevent arrhythmia formation. Both medications and surgical sympathectomy have been used to abate the increased sympathetic activity following MI and refractory ventricular arrythmias (Fukuda et al., 2015; Hadaya and Ardell, 2020), with sympathectomies, a procedure that removes the lower third of the stellate ganglia through the T4 level of the paravertebral chain, becoming increasingly common for the management of chronic ventricular tachycardia (Dusi et al., 2022; Shivkumar and Zipes, 2022). Bioelectronic nerve blocks have been shown to be able to produce similar effects to sympathectomy, but in a temporary and reversible manner (Buckley et al., 2017; Chui et al., 2017; Hadaya et al., 2022; Vrabec et al., 2024). By modulating the neural inputs to the heart, we aim to create a closed-loop therapy that can predict the onset of arrhythmias before they happen, so that there is no need to apply a cardioversion shock to the patient. Future works investigating this therapy should investigate long-term efficacy and safety of this treatment in awake, mobile animals, and compare the effects of unilateral block against bilateral block of the sympathetic chain. Closed-loop control of this electrical nerve block could provide an “on-demand sympathectomy,” blocking sympathetic activity only when it is needed, once adverse changes in cardiac function are predicted or seen. This could limit the frequency and severity of the side effects from surgical sympathectomies, improving patient outcomes while not compromising on the efficacy of the treatment.

## Data Availability

The raw data supporting the conclusions of this article will be made available by the authors, without undue reservation.
