# Supplementary material for: Closed-loop bioelectronic block of stellate ganglia reduces neurotransmitter release and improves recovery following programmed ventricular pacing
Source: Front Bioeng Biotechnol. 2026 Aug 10;14:1828505. doi: 10.3389/fbioe.2026.1828505 (PMC13500574; doi:10.3389/fbioe.2026.1828505)
Supplement: Supplementary file 1 [file DataSheet1.docx]

Supplementary Material

# 1 Methods

## 1.1 Chronic Infarction Procedure

Six weeks prior to terminal experiments, 5 pigs were subjected to a chronic myocardial infarction (MI) preparation. Animals were initially sedated with an intramuscular injection of midazolam (1 mg/kg) and ketamine (10 mg/kg). After anesthesia was induced, the animals were endotracheally intubated and ventilated (tidal volume 300-450mL, rate 10-12 breaths per minute), and anesthesia was maintained with inhaled isoflurane (1-4%). Additional intramuscular injections of carprofen (4.4 mg/kg) and buprenorphine (0.02 mg/kg) were administered as perioperative analgesics. Anesthesia depth was assessed by the corneal blink reflex, muscle tone and fasciculation, 12-lead electrocardiogram (EKG), oxygen saturation, and end-tidal carbon dioxide; anesthetic delivery was adjusted to ensure the animals remained in the appropriate anesthetic plane for the entirety of the procedure.

Amiodarone (1.5 mg/kg, intramuscular) and lidocaine (2 mg/kg, intravenous) were given for ventricular arrythmia prophylaxis, and intravenous heparin (5000 units) was given to prevent excessive coagulation. Body temperature was maintained using a forced-air normothermia system, and 0.9% NaCl (5-6 mL/kg/hour) was given to mitigate excessive fluid loss.

Either the right or left femoral artery was catheterized with a vascular access sheath. The left coronary artery was cannulated with an AL1.0 guide catheter (Medtronic, Minneapolis, Minnesota, USA), and then a 0.014 inch guidewire (Balance Middleweight Universal, Abbott Laboratories, Chicago, IL, USA) was passed through the catheter. After removing the guide catheter, an angioplasty balloon catheter was passed over the guidewire, and inflated in the mid-left anterior descending (LAD) coronary artery at the level of the second diagonal branch. Polystyrene microspheres (90 μm mean diameter) in suspension (Polysciences Inc., Warrington, PA, USA) were diluted with normal saline (2.25 ml diluted to 6 ml with 0.9% NaCl) and were injected to embolize the LAD distal to the balloon.

Following embolization, ventricular repolarization changes were observed via hyperacute T waves, T wave inversion, or ST segment elevation on the 12-lead EKG. If an animal developed ventricular tachycardia or ventricular fibrillation, external electrical defibrillation and standard cardiopulmonary resuscitation would be performed as needed until the arrhythmia resolved. Following resuscitation, intravenous epinephrine (0.01 mg/kg) would be administered every 3 to 5 minutes, and the antiarrhythmic drugs would be given again (intravenous amiodarone 1.5 mg/kg and lidocaine 2 mg/kg). Following embolization, the catheters were removed, and the animal was extubated and allowed to regain consciousness under supervision.

Following the infarction preparation, animals were redosed with intramuscular carprofen (4.4 mg/kg) daily to minimize pain. In the six weeks between the infarct and the terminal experiment, the animal was monitored daily for signs of pain or distress.

## 1.2 Terminal Surgical Procedure

The terminal experiments for both the healthy control animals and the chronically infarcted animals followed the same procedure. Initial anesthesia induction, ventilation, and isoflurane maintenance followed a similar protocol as described above; for the terminal experiments, intramuscular tiletamine-zolazepam (4-6 mg/kg) was given to induce anesthesia, and intravenous fentanyl citrate (20 mg/kg, intravenous) was given as a stronger analgesic.

The femoral arteries on each side were catheterized with vascular access sheaths. These sheaths were used to administer intraoperative fluids, monitor arterial blood gases, and provide an access point to place a solid-state blood pressure catheter (Mikro-Tip Model SPR-350, Millar Instruments, Houston, TX, USA) in the left ventricle. The arterial blood gases were monitored hourly to ensure that ventilation and fluid delivery were sufficient; ventilation adjustments were made or sodium bicarbonate boluses were administered to ensure that the animal’s acid/base parameters remained normal throughout the experiment. The right jugular vein was accessed in a similar manner to deploy the right ventricular pacing catheter mid-septum (Boston Scientific, Marlborough, MA, USA).

A midline sternotomy was performed to open the thoracic cavity and expose the heart. The parietal pleura was detached from the thoracic wall to access the right paravertebral chain. The chain was isolated across the T1-T4 spinal levels; a stimulating bipolar platinum nerve cuff (MicroProbes for Life Science, Gaithersburg, MD, USA) was placed on the chain between the T2 and T3 ganglia, and a custom electrode as part of a carbon separated interface nerve electrode (CSINE) (Vrabec et al., 2019a) was placed between T1 and T2. The return electrode for the CSINE was either a stainless steel needle electrode in the chest wall near the nerve, or another CSINE saline channel of the side of the block cuff electrode that faced away from the nerve. A diagram of this setup can be seen in the inset in Figure 1.

After the surgical setup was performed, the anesthesia was transitioned from inhaled isoflurane to intravenous alpha-chloralose. Alpha-chloralose was chosen as it does not depress cardiac or other autonomic reflexes. A bolus of 50mg/kg of alpha-chloralose was given before isoflurane was turned off. After the bolus, alpha-chloralose was infused intravenously starting at 10-20 mg/kg/hr, with this rate being adjusted as needed throughout the experiments to maintain appropriate anesthetic depth.

## 1.3 Euthanasia

After completion of the terminal experiments, animals were euthanized in accordance to IACUC protocol 2016-084. Briefly, once data collection had finished, the rate of anesthesia was doubled, and ventricular fibrillation was induced with an electric current applied to the ventricle surface. Ventilation was stopped following fibrillation induction. The animal was monitored for a minimum of 10 minutes following fibrillation, and euthanasia was confirmed by a lack of heartbeat and blood pressure pulses.

## 1.4 Measurement of Hemodynamic and Electrophysiological Data

### 1.4.1 Intracardiac Echocardiography

In chronically infarcted animals, an intracardiac echocardiograph was taken prior to sternotomy. An ultrasonic catheter (ViewMate ICE Catheter, Abbott Cardiovascular, Plymouth MN, USA) was inserted through a femoral access sheath, and mapping of cardiac contraction was performed. This was used to analyze the size of the scar tissue formed as a result of the infarct.

### 1.4.2 Hemodynamic Measurements

In addition to the solid-state catheter measuring left ventricular pressures, a fluid pressure transducer was attached to the vascular access sheath in a femoral artery; this provided arterial blood pressure recordings to complement the ventricular pressure recordings.

### 1.4.3 Measurement of NE and NPY

Briefly, cardiac catecholamines were measured using fast scanning cyclic voltammetry (FSCV) and capacitive immunoprobes (CI) used to measure norepinephrine (NE) and neuropeptide Y (NPY), respectively.

#### 1.4.3.1 Instrumentation

Custom equipment to perform these measurements was designed in-house and was based on an NPI VA-10M, 4-channel amplifier (NPI Electronic, Tamm, Germany). A head stage unit based on the NPI HS-VA-04 (NPI Electronic, Tamm, Germany) was also developed to connect the 4 measurement probes to a single pair of reference and counter electrodes. A 1 MΩ feedback resistor was used for all measurements in these experiments. In this preparation, the sensing electrodes were long, platinum electrodes (described below); as such, a low-resistance feedback circuit was required to deliver enough charge to the higher-capacitance electrodes to provide sufficiently fast voltage slew rate. For FSCV measurements analyzed in real-time for use in the closed-loop controller, different equipment was used, as described in the Closed-Loop Controller section below.

A three-electrode measurement was used for both techniques consisting of a working electrode embedded in the myocardium, and reference and counter electrodes placed in the chest wall. For the FSCV measurements, stainless steel needles were used for the reference and counter electrodes, while the CI measurement used Ag/AgCl disk electrodes (World Precision Instruments, Sarasota, FL, USA) placed into exposed intercostal muscle tissue on the caudal end of the sternotomy. In both the FSCV and CI measurements, the counter electrode was electrically connected to the chassis and earth grounds of the amplifier. The electrical potential between the working and reference electrode was controlled using custom software through the digital-to-analog output channels of a HEKA LIH 8+8 AD/DA device (HEKA Elektronic, Holliston, MA, USA). Electrodes were 30 cm of 127 µm diameter PFA-coated Pt wire (A-M Systems, Sequim, WA, USA). One end of the wire had the insulation stripped and was crimped and soldered to a gold pin to connect with the amplifier head stage; the pin connection was also soldered to provide mechanical stabilization. The other end was threaded through a 23-gauge hypodermic needle, and a ~5 mm barb was bent into the end. The needle was used to embed the electrode into the myocardium, the needle was then withdrawn and the barb secured the electrode in place; electrical measurements take place at the uninsulated circular cross section at distal tip of the wire electrode. More detailed instructions on FSCV electrode fabrication have been given previously (Chan et al., 2020).

For FSCV NE measurements, the electrodes were cycled through a sawtooth command potential waveform (described below). The reference potential was provided by a reference and ground electrode circuit with those electrodes implanted in the chest wall as described above. The current required to follow the command potential was measured to provide signal in each channel. NE signal was determined by subtracting background circuit capacitance from the recorded voltammograms and measuring the remaining current at the peak oxidation potential for NE.

CI electrodes to measure NPY initially follow the same fabrication steps as the FSCV electrodes. Following the threading and barbing of the electrode, polydopamine was electrodeposited on the exposed tip of the wire electrode. The coated electrode tips were then placed in a 20:1 solution of phosphate buffered saline (PBS; pH 7.4) to monoclonal anti-NPY antibody (Abcam, Cat # ab112473 clone 8, Cambridge, MA, USA). The electrodes were left in the solution for at least one hour at room temperature to give ample time for the anti-NPY antibody to bind to the polydopamine on the probe tip. More detailed instructions on CI electrode fabrication have been given previously (Kluge et al., 2021). CI electrodes were deployed as described for FSCV electrodes above. CI electrodes were cycled through a step potential command potential protocol (described below). Binding NPY to the electrode tip alters the total circuit capacitance by altering the Helmholtz layer and increases measured charge current proportional to NPY (Kluge et al., 2021).

In total, 8-9 electrodes were placed in the left ventricular tissue (4-5 FSCV, 4 CI). Two of each type were placed in the lateral aspect the left ventricle, towards the base of the heart, and two were placed more apical, in the medial region of the left ventricle (Figure 1). In chronically infarcted animals, the medial probes were placed in the border zone around the infarction scar tissue. If space and animal condition allowed it, a third FSCV electrode was placed in the lateral region for

#### 1.4.3.2 Acquisition and Analysis

The software of for controlling the voltage profile and measuring current changes was written in-house in IGOR Pro (v. 8.04 WaveMetrics, Lake Oswego, OR, USA). The LIH 8+8 issued a command potential to the VA-10M amplifier, which drove the potential at the electrode with a gain of 5x. The voltage profile differed between the FSCV and CI measurements, and is described below. The LIH 8+8 was also responsible for recording current data from the amplifier. Since the voltage profiles for FSCV and CI were different, we employed two identical sets of computer-AD/DA-amplifier systems concurrently for these two data sets. All data recorded were sampled at 10 kHz after being filtered by an 8-pole analog Bessel filter with a cutoff frequency of 1 kHz.

For FSCV measurements, a triangular voltage waveform was delivered to the measurement electrodes, and the resultant current was measured. The voltage waveform oscillated between -0.5 V and +1.2 V, with a slope of 12 V/s in both directions; this gives an effective sample rat of ~3.5 Hz. Basal absolute norepinephrine concentrations were estimated by subtracting an ohmic trajectory between the currents at -0.5 V and +1.2 V; NE was estimated by comparing the resultant currents measured at the peak NE oxidation potential (Chan et al., 2020). Changes in norepinephrine were measured by first subtracting an averaged current profile from the 10 cycles preceding the programmed pacing; this removed effects from the intrinsic capacitance of the electrode and the basal NE concentration, and any change in current could be attributed change in the amount of norepinephrine that was being oxidized. This protocol was described in more detail previously (Chan et al., 2020).

For CI measurements, the probe potentials were cycled through an asymmetric, discontinuous square wave. Each cycle was 80 ms long, composed of four 20 ms steps at 0 mV, 100 mV, 0 mV, and -5 mV. As more NPY binds to the probe antibodies, double-layer capacitance at electrode-electrolyte interface also increases; this change in capacitance is measured as the current charge required to bring the probe from 0 mV to 100 mV. During the -5 mV step, the NPY is electrostatically repelled from the probe and reopens the antibody binding sites for a subsequent round of measurement. Repeats of the cycle every 80 ms provides electrode-NPY binding as a function of time.

#### 1.4.3.3 Norepinephrine Selectivity

Previous experiments performed *in vivo* have shown that our FSCV measurements correspond to norepinephrine, and not other catecholamines, as shown in Figure S1. In the top portion, NE was infused into the heart as a known analyte, and an increase in oxidation current is detected. In the same animal, vagus nerve stimulation (parasympathetic activation), followed by bilateral stellate ganglia (BSG) stimulation at 10 Hz was delivered. At the same oxidation potential, a lowering of current is observed from VNS, and an increase is seen with BSG stimulation. This, along with the information that NE is the primary neurotransmitter in the heart, indicates that we are in fact measuring norepinephrine release.

While not ideal for differentiating between catecholamines, the lower scan rates of our measurements still create enough separation to selectively differentiate measurements from other neurotransmitters. Figure S2 is an example of FSCV performed *in vitro* at 12 V/s comparing the oxidation potentials between norepinephrine, dopamine, and serotonin at 250 μM in a flow chamber. While dopamine and norepinephrine significantly overlap, the serotonin peak is small and at a higher potential (400mV vs ~0V for NE), selectivity from non-catecholamines.

**Figure S1:** This plot demonstrates a positive control for the selectivity of our measurement for norepinephrine. The upper pair of plots show the change in current from infusion of norepinephrine, while the bottom pair of plots show a change in current from neural stimulation. The oxidation potential for the infused norepinephrine is the same as that as is seen in the sympathetic stimulation, indicating that we are measuring norepinephrine.

Beyond this, our selectivity benefits from both our anatomical location of measurement and subtraction of the baseline signal. This is comparable to measurements taken in the brain, where neurotransmitters are often identified via anatomical location or by the stimulus they respond to, as opposed to differences in oxidation potential (particularly to separate norepinephrine from dopamine). This method of selectivity is sufficient despite non-zero concentrations of other electrically active neurotransmitters in the brain and cerebrospinal fluid.

**Figure S2:** Voltammograms from a 12V/s FSCV scan with electrodes in 250 μM flow dishes of norepinephrine, dopamine, and serotonin.

Furthermore, while other neurotransmitters are present and may change following MI, they are not expected to meaningfully change on the time scales we are measuring. As norepinephrine is the primary sympathetic neurotransmitter (with the non-electroactive acetylcholine being the primary parasympathetic neurotransmitter), we expect any acute changes (i.e. in seconds to minutes following the start of pacing) to the oxidation current from the pacing protocol to be purely from norepinephrine, as opposed to chronic changes over the span of hours to days for other neurotransmitters. Baseline subtraction for each trial individually ensures that any medium-term changes are removed before the measuring the change in current from programmed pacing.

## 1.5 Block Threshold Testing

### 1.5.1 Sympathetic Stimulation

As described in the terminal surgical procedure, a bipolar stimulation electrode was placed on the paravertebral chain at the T2-T3 spinal level. Monophasic current-controlled stimulation with a pulse width of 4 ms was applied at 4 Hz from an S88 stimulator with a PSIU-6 linear current-controlled isolation unit (both Grass Instruments, Warwick, RI, USA). A modified binary search was performed to find the amplitude of stimulation that would raise the heart rate 15% in 20 seconds. This current was tripled, and used to test for block efficacy; if the tripled current exceeded 15 mA or caused a dangerously high heart rate increase, the 10%-increasing current was used.

**Figure S3:** This plot shows the change in heart rate for three separate block thresholding attempts. The unblocked T2-T3 stimulation causes the highest change in heart rate (red). A sub-threshold block attenuates this increase but does not remove it (yellow). The block threshold is able to completely abolish the heart rate change from stimulation (green).

### 1.5.2 Direct Current Electrical Nerve Block

Direct current (DC) electrical nerve block (ENB) was applied to the paravertebral chain at the T1-T2 spinal level from a current source (Keithley 2450, Cleveland, OH, USA) via a carbon separated-interface nerve electrode (CSINE) (Vrabec et al., 2019b). The CSINE uses a high-capacitance slurry that allows for the safe injection of DC without evolving harmful chemical reactants. The block threshold (BT_120_) was defined as the minimum cathodic current that would completely abolish the response from the tripled T2-T3 stimulation. A trial with no block was run, and 15 seconds of the stimulation were given; the resultant peak in heart rate was noted. After the heart rate recovered and stabilized, a blocking current was delivered for 2 minutes, followed by 15 seconds of stimulation while the block was left on. The block and stimulation were turned off together. If a change in heart rate was seen with the stimulation, the current was increased; if the stimulation produced no change in heart rate, the block was decreased. This was repeated until the minimum current to completely block was found, measured to the nearest 0.5 mA. An example of block thresholding can be seen in Figure S3.

A: Normalized Norepinephrine Membership

B: Norepinephrine Slope Membership

C: Output Current Change Membership

D: Control Diagram

**Figure S4**: The above plots show the membership functions and the controller flow diagram for the fuzzy logic controller used in these experiments. Subplot A shows the input membership functions for the normalized change in NE as a fraction of the setpoint (i.e. an increase of 75 nA with a setpoint of 50 nA would be 150% of the setpoint). Subplot B shows the input membership function for the slope of the normalized NE current. Subplot C shows the output membership functions for the change in blocking current output, as a change proportional to block threshold. Subplot D shows the controller diagram for these experiments.

### 1.5.3 Closed-loop Controller

Unlike our previous work that used proactive, static blocking currents to mitigate catecholamine release (Vrabec et al., 2024), this study measured the release of norepinephrine in real-time, and turned on block retroactively once the change in norepinephrine hit a predefined threshold.

#### 1.5.3.1 Controller Design

Figure S4 shows the overall control diagram for the fuzzy logic system, with subplot D showing the controller diagram. For these experiments, the controller was designed to begin applying DC ENB once the measured NE current reached a predefined threshold. In Figures S4A and S4C, this corresponds to the membership function expressed as the red line. When the measured NE was below the threshold, the blocking current was reduced (Figure S4A and S4C green line). If the NE level was seen as high, but decreasing, the increase in blocking current was attenuated so as to dampen the potential for oscillations (Figures S4A and S4C dashed blue line, and Figure S4B).

#### 1.5.3.2 Instrumentation

The norepinephrine signal was recorded in a similar manner to what is described above, but with different instrumentation. The probe in a lateral/remote zone of the left ventricle with the highest NE release chosen for the controller for all experiments. For the real-time controller input signal, an off-the-shelf BioLogic Sp-300 (BioLogic, Seyssinet-Pariset, FR) potentiostat was used. Using the same electrodes as described above, the SP-300 also used a 3-electrode setup, with the main differentiator being that the counter-electrode was not hooked up to ground and was instead operated in a floating mode (preliminary work showed that this reduced interference between the two systems and the electrophysiological recordings). Using the cyclic voltammetry firmware, a triangle wave was cycled between -0.5 V and 1.2 V, at 6 V/s (~1.7647 Hz). Data were recorded at 500 Hz and were sent via a USB connection to a computer running custom LabVIEW software (NI, Austin, TX, USA). This software analyzed the FSCV data and updated the controller output at a rate of 2 Hz. The blocking current was applied to the paravertebral chain at the T1-T2 level via a CSINE, described above. The same Keithley 2450 received amplitude commands from the controller software and applied the corresponding cathodic current to the nerve. The controller had hard limits at 0% BT_120_ (no current) and 150% BT_120_.

### 1.5.4 Data Collection and Analysis

Real-time data analysis followed the following steps: Data sent to the LabVIEW program were first organized to ignoring the last cycle if it was not completed yet. A baseline cycle was selected, typically the latest cycle immediately preceding the programmed pacing that did not have any interference (i.e. EKG noise). The current profile of the baseline cycle was subtracted from every other cycle, resulting in a plot of difference in current from the baseline cycle, ΔI. Durning the preliminary unblocked trial, a potential was selected that showed the strongest norepinephrine oxidation signal (0 mV to 250 mV); this potential was used throughout a full set of experiments. The controller took the current readings at the oxidation potential for each cycle and plotted the change in current against time. This signal was fit to a sigmoidal curve to account for oxidation of the electrode itself to come to equilibrium at the start of each trial. This curve was fit for the two minutes of data preceding the programmed pacing, not changing once the pacing had begun. This fit curve was subtracted from the ΔI measurements. These data were further filtered by moving 11-point median filter to remove any spurious points, such as those caused by EKG interference. This resulting smoothed current reading was then sent to the controller inputs, along with the point-to-point slope of this measurement.

### 1.5.5 Data extraction and post-hoc analysis

All the data presented herein are from post-hoc analysis of the recordings. Real-time data calculation was used to control the block during the experiment, but post-hoc analysis was used to determine statistical significance.

#### 1.5.5.1 Norepinephrine Data

The norepinephrine data from both instruments were analyzed using similar methods. After subtracting a baseline cycle, the current profiles at the NE oxidation potential were extracted; the oxidation potential was chosen during the unblocked trial, and remained consistent for a whole experimental set, for both setups. The resultant current profile was fit to curve and subtracted remove electrode effects during the baseline portion of the trial, with the controller data using a sigmoidal fit and the global data using an exponential fit. This process is described in more detail in previous work (Chan et al., 2020). Data were then smoothed to remove noise and interference, the controller with a sliding 11-point median filter, and the global data was left unfiltered due to the better signal-to-noise ratio of the custom amplifier. The primary difference between the controller NE data analysis and the global NE data analysis is that the controller data is normalized within a set (normalized to the unblocked trial), whereas the global data is reported without normalization.

#### 1.5.5.2 Neuropeptide Y Data

The NPY CI data of each experiment were acquired on the separate setup simultaneously with the FSCV data. For each experiment the peak capacitive current of each cycle was plotted and used for further analysis. The CI current was baseline corrected by fitting and subtracting the first 120 second CI data. The CI data were further smoothed by a 5 second windows. The resulting CI currents were analyzed and reported as the peak CI current during the pacing and the settling CI current (at the end of the pacing).

#### 1.5.5.3 Hemodynamic Data

Data was acquired using Cambridge Electronic Design System Power 1401 A/D and Spike 2 software (Spike2^TM^, Cambridge Electrical Design, Cambridge U.K.) HR was calculated using the peak LVP waveform. The maximum rate of change of LV pressure (LV dP/dt_max_) was used to assess LV contractility, while the minimum rate of change of LV pressure (LV dP/dt_min_) was used to assess lusitropy.

# 2 Results

## 2.1 Perturbation Consistency

To ensure that our programmed pacing protocol was consistent between trials, we measured the time it took the pacing to produce a 33% increase in the NE levels; this measurement is applied prior to the application of block in each trial. This measurement showed no significant differences seen between any pairs, either at a set level between healthy and infarcted animals, or within a model between block setpoints. This indicates that the pacing had a similar effect for each trial, affirming the repeatability of the protocol and the independence of the trials. Summary statistics can be seen in Table S1.

## 2.2 Ancillary NE Data Measurements

| Rise Time (s) | Healthy | | | MI | | |
| --- | --- | --- | --- | --- | --- | --- |
|  | Median | Mean | Std. Dev. | Median | Mean | Std. Dev. |
| No Block | 28.5 | 30.9 | 15.1 | 31.9 | 35.0 | 16.3 |
| 33% NE Reduction | 40.4 | 46.6 | 31.6 | 40.9 | 54.2 | 38.8 |
| 66% NE Reduction | 44.3 | 46.5 | 26.8 | 52.8 | 48.8 | 9.6 |

**Table S1**: Median, mean, and standard deviations for the time it took the programmed pacing to elicit a 33% increase from baseline. Although varied, there is not a significant difference between the trial types, or between healthy or MI animals, indicating that the effect of programmed pacing on NE release was generally consistent between trials.

### 2.2.1 Peak NE Current

The top portion of Table S2 shows the maximum change in NE current as a result of programmed pacing. Between each combination of pairs between controller setpoint (no block, 33%, 66%) and electrode region, there were no significant differences seen. Between healthy and MI animals, only two significantly different pairs were seen (Medial/Border Zone, unblocked trials p = 0.0346; Medial/Border Zone, 33% Reduction trials p = 0.0477). The lack of differences between setpoints indicates that the controller had poor global control, despite seeing significant differences in regional control.

|  |  |  | Healthy Animals | | | MI Animals | | |
| --- | --- | --- | --- | --- | --- | --- | --- | --- |
|  |  |  | Median | Mean | Std. Dev. | Median | Mean | Std. Dev. |
| Max NE (nA) | No Block | Lateral/Remote | 60.6 | 96.1 | 309.4 | 134.0 | 173.4 | 257.7 |
|  |  | Medial/Border | 122.6 | 108.7 | 96.3 | 128.3 | 166.6 | 184.2 |
|  | 33% NE Reduction | Lateral/Remote | 134.3 | 159.9 | 246.7 | 21.5 | 54.2 | 157.8 |
|  |  | Medial/Border | 82.3 | 105.3 | 259.9 | 100.3 | 150.8 | 137.5 |
|  | 66% NE Reduction | Lateral/Remote | 127.4 | 194.3 | 283.5 | 99.0 | 71.4 | 95.2 |
|  |  | Medial/Border | 64.5 | 88.4 | 181.3 | 88.9 | 94.0 | 187.8 |
| Settling NE (nA) | No Block | Lateral/Remote | 21.7 | 62.4 | 176.9 | 77.7 | 110.9 | 153.2 |
|  |  | Medial/Border | 18.3 | 31.3 | 59.9 | 105.0 | 132.9 | 138.7 |
|  | 33% NE Reduction | Lateral/Remote | 106.1 | 131.3 | 170.0 | 7.3 | 39.9 | 109.4 |
|  |  | Medial/Border | 18.0 | 60.2 | 164.3 | 102.7 | 176.2 | 238.9 |
|  | 66% NE Reduction | Lateral/Remote | 46.4 | 67.9 | 164.3 | 59.8 | 47.4 | 73.4 |
|  |  | Medial/Border | 16.6 | 17.7 | 103.5 | 52.0 | 65.6 | 151.0 |

**Table S2**: Median, mean, and standard deviation of the maximum NE current during programmed pacing, and the mean NE current during the last 30s of programmed pacing. In general, few significant differences were seen between any groups for any measurement.

### 2.2.2 Settling NE Current

The bottom portion of Table S2 shows the mean change in NE current during the last 30 s of programmed pacing, deemed the settling value. As for the maximum value, between each combination of pairs between healthy/MI animals, controller setpoint, and electrode region, no significant differences were seen. In addition to the max currents, the lack of differences between setpoints in the mean currents indicates that the controller had poor global control, despite seeing significant differences in regional control.

Due to the electrode with the highest measured NE release being used for the controller input, the electrodes not used in the controller had a bias towards having lower and/or less responsive NE measurements. For the unblocked trials in the healthy animal, the medial region showed a significant difference from the baseline measurement (p=0.0152), while the lateral region did not (p=0.3054). In the MI animals, both the border (p=0.0049) and the remote (p=0.0093) mean NE currents differed from the baseline. In the 33% reduction trials, only two of the 4 regions showed any difference from baseline (healthy lateral p=0.0210; healthy medial p=0.1726; MI remote p=0.2661, MI border p=0.0034). For the 66% reduction trials, no zone showed a significant difference between the mean value and the baseline measurement, indicating that no change in NE was detected throughout the duration of the trial (healthy lateral p=0.2754; healthy medial p=0.4238; MI remote p=0.0742, MI border p=0.2031).

## 2.3 NPY Data

### 2.3.1 Peak NPY data

The top portion of Table S3 shows the maximum change in NPY current as a result of programmed pacing. Between each combination of pairs between animal model, controller setpoint, and electrode region, there was only one significantly different pair seen (MI animals, no block, remote/border p=0.0376

|  |  |  | Healthy Animals | | | MI Animals | | |
| --- | --- | --- | --- | --- | --- | --- | --- | --- |
|  |  |  | Median | Mean | Std. Dev. | Median | Mean | Std. Dev. |
| Max NPY (nA) | No Block | Lateral/Remote | 17.1 | 31.4 | 34.8 | 28.6 | 38.0 | 35.3 |
|  |  | Medial/Border | 16.0 | 12.2 | 7.5 | 6.8 | 10.9 | 14.0 |
|  | 33% NE Reduction | Lateral/Remote | 18.5 | 19.2 | 12.4 | 23.4 | 21.9 | 17.1 |
|  |  | Medial/Border | 18.9 | 19.3 | 10.8 | 11.8 | 11.1 | 14.6 |
|  | 66% NE Reduction | Lateral/Remote | 24.1 | 24.4 | 15.1 | 23.7 | 24.1 | 14.0 |
|  |  | Medial/Border | 19.8 | 22.6 | 13.8 | 8.4 | 15.8 | 19.8 |
| Settling NPY (nA) | No Block | Lateral/Remote | 13.9 | 22.3 | 35.7 | 17.5 | 27.5 | 36.3 |
|  |  | Medial/Border | 6.7 | -0.5 | 16.4 | -7.5 | -11.7 | 28.8 |
|  | 33% NE Reduction | Lateral/Remote | 12.3 | 5.2 | 27.4 | 8.8 | 7.6 | 23.7 |
|  |  | Medial/Border | 10.8 | -2.8 | 39.4 | -18.3 | -8.6 | 24.9 |
|  | 66% NE Reduction | Lateral/Remote | 8.3 | 3.6 | 14.3 | 12.3 | 13.1 | 18.4 |
|  |  | Medial/Border | 0.6 | -3.2 | 18.6 | 3.0 | 1.6 | 28.7 |

**Table S3**: Median, mean, and standard deviation of maximum NPY current during programmed pacing, and the mean NPY current during last 30s of programmed pacing. Few significant differences were seen between any groups for any measurement due to overall low NPY release during these experiments, but some reduction in spread was seen in the blocked trials compared to the unblocked trials.

In the lateral region of healthy animals, the unblocked trials had a significantly greater standard deviation (superiority test for the ratio of standard deviations, ratio = 1.0) than the 66% reduction trials (p=0.0446). There was also a strong but no significant increase in the standard deviation from the 33% Reduction trials to the unblocked trials in the same region (p=0.0595). A similar effect was seen in the remote region of infarcted animals, with the unblocked trials having a significantly greater standard deviation than both the 33% reduction trials (p=0.0067) and the 66% reduction trials (p=0.0045).

### 2.3.2 Settling NPY Current

The bottom portion of Table S3 shows the settling value for NPY current, as measured by the mean of the last 30 s of programmed pacing. Similar to the maximum currents, there was only one significant difference seen between each combination of pairs between animal model, controller setpoint, and electrode region (MI animals, no block, remote/border p=0.0109).

In the lateral region of healthy animals, the unblocked trials had a significantly greater standard deviation (superiority test for the ratio of standard deviations, ratio = 1.0) than the 66% reduction trials (p=0.0331). A similar effect was seen in the remote region of infarcted animals, with the unblocked trials having a significantly greater standard deviation than the 66% reduction trials (p=0.0239).

It is worth noting that NPY release was low overall in these experiments. According to a Wilcoxon signed-rank test, only the unblocked remote zone in the MI animals had a settling current that was significantly different from the baseline, pre-pacing levels. (p = 0.0067).

# 3 Supplementary Material References

CHAN, S. A., VASEGHI, M., KLUGE, N., SHIVKUMAR, K., ARDELL, J. L. & SMITH, C. 2020. Fast in vivo detection of myocardial norepinephrine levels in the beating porcine heart. *Am J Physiol Heart Circ Physiol,* 318**,** H1091-H1099.

KLUGE, N., DACEY, M., HADAYA, J., SHIVKUMAR, K., CHAN, S. A., ARDELL, J. L. & SMITH, C. 2021. Rapid measurement of cardiac neuropeptide dynamics by capacitive immunoprobe in the porcine heart. *Am J Physiol Heart Circ Physiol,* 320**,** H66-H76.

VRABEC, T., BENDER, S., CHAN, S. A., CHA, S., HARIDAS, S., HANNA, P., AJIJOLA, O. A., SHIVKUMAR, K., SMITH, C. & ARDELL, J. L. 2024. Bioelectronic block of stellate ganglia mitigates pacing-induced heterogeneous release of catecholamine and neuropeptide Y in the infarcted pig heart. *J Physiol,* n/a.

VRABEC, T. L., WAINRIGHT, J. S., BHADRA, N., SHAW, L., KILGORE, K. L. & BHADRA, N. 2019a. A carbon slurry separated interface nerve electrode for electrical block of nerve conduction. *IEEE Transactions on Neural Systems and Rehabilitation Engineering.* IEEE.

VRABEC, T. L., WAINRIGHT, J. S., BHADRA, N., SHAW, L., KILGORE, K. L. & BHADRA, N. 2019b. A Carbon Slurry Separated Interface Nerve Electrode for Electrical Block of Nerve Conduction. *IEEE Trans Neural Syst Rehabil Eng,* 27**,** 836-845.
